# Cordycepin enhances the chemosensitivity of esophageal cancer cells to cisplatin by inducing the activation of AMPK and suppressing the AKT signaling pathway

**DOI:** 10.1038/s41419-020-03079-4

**Published:** 2020-10-16

**Authors:** Ying Gao, Dan-Lei Chen, Mi Zhou, Zhou-san Zheng, Mei-Fang He, Sheng Huang, Xiao-Zhong Liao, Jia-Xing Zhang

**Affiliations:** 1 Department of Oncology, the First Affiliated Hospital of Sun Yat-sen University, Guangzhou, 510080 China; 2 Department of Orthopaedics, the First Affiliated Hospital of Nanchang University, 330006 Nanchang, China; 3 Department of Oncology, the First Affiliated Hospital of Guangzhou University of Chinese Medicine, Guangzhou, 510405 China

**Keywords:** Cancer, Oesophageal cancer

## Abstract

Although cisplatin (cDDP), is a first-line chemotherapy drug for esophageal cancer, it still has the potential to develop drug resistance and side effects. There is increasing evidence that cordycepin can work synergistically with other chemotherapy drugs. Therefore, we investigated whether combination therapy of cordycepin and cDDP may enhance the therapeutic effect of cDDP. We performed a series of functional tests to study the effect of medical treatment on esophageal cancer cells. We then used GO analysis to examine the pathways affected by treatment with cordycepin and cDDP. Next, we observed changes in the abundance of the selected pathway proteins. The in vivo animal model supported the results of the in vitro experiments. Co-treatment with cordycepin and cDDP inhibited cell growth, migration, and metastasis, as well as induced apoptosis. Cordycepin was found to effectively enhance activation of AMPK and inhibited activity of AKT. In all treatment groups, the expression levels of p-PI3K, p-Akt, p-p70S6K, Caspase-3, and Bcl-2 were significantly reduced, while the expression levels of p-AMPK, cleaved Caspase-3, and Bax increased, and the total levels of Akt, PI3K, and p70S6K levels remained unchanged. Overall, cordycepin was found to enhance the chemical sensitivity of esophageal cancer cells to cisplatin by inducing AMPK activation and inhibiting the AKT signaling pathway. Combination therapy of cordycepin and cisplatin represent a novel potential treatment of esophageal cancer.

## Introduction

Esophageal cancer is one of the most common types of cancer in the world^1^, and only 20% of patients have esophageal cancer that can be treated with surgery. Other treatments for esophageal cancer include radiotherapy and chemotherapy^2^. However, accumulating evidence shows that esophageal cancer has increased resistance to conventional chemotherapy drugs. Therefore, it is important to discover a novel and effective drug for the treatment of esophageal cancer.

Cordycepin, a nucleoside analogue, is the main biologically active ingredient found, isolated, and purified in *Cordyceps militaris*. It has effects on several biological processes including regulation of the inflammatory response^3^, platelet aggregation^4^, and steroid production^5^. It is reported that cordycepin is also involved in regulating protein synthesis and cell adhesion^6^. In addition, cordycepin is thought to play an essential role in the inhibition of cell proliferation and invasion and tumor metastasis through various signaling pathways^7–10^.

Cisplatin (cDDP) is one of the most effective chemotherapy treatments for adjuvant or neoadjuvant treatment of testicular, ovarian, head, and neck tumors, and various cancers, including esophageal cancer^11^. The main anti-cancer mechanism of cDDP is through its interaction with purine bases in DNA, leading to the formation of DNA-protein and DNA-DNA inter- and intra-strand cross-links that result in tumor cell proliferation inhibition and apoptosis^11^. Recently, there has been increasing evidence that the cisplatin-mediated activation of AMP-activated protein kinase (AMPK) is involved in the apoptosis of esophageal cancer cells through mammalian target of rapamycin (mTOR)^12,13^. Meanwhile, through the combined treatment of cDDP and other drugs, such as Oridoni^14^, inhibition of mTOR expression can enhance the effects of cytotoxicity and cellular apoptosis. However, the role of cDDP and cordycepin in combination therapy for treatment of esophageal cancer is largely unknown.

**Fig. 1:**
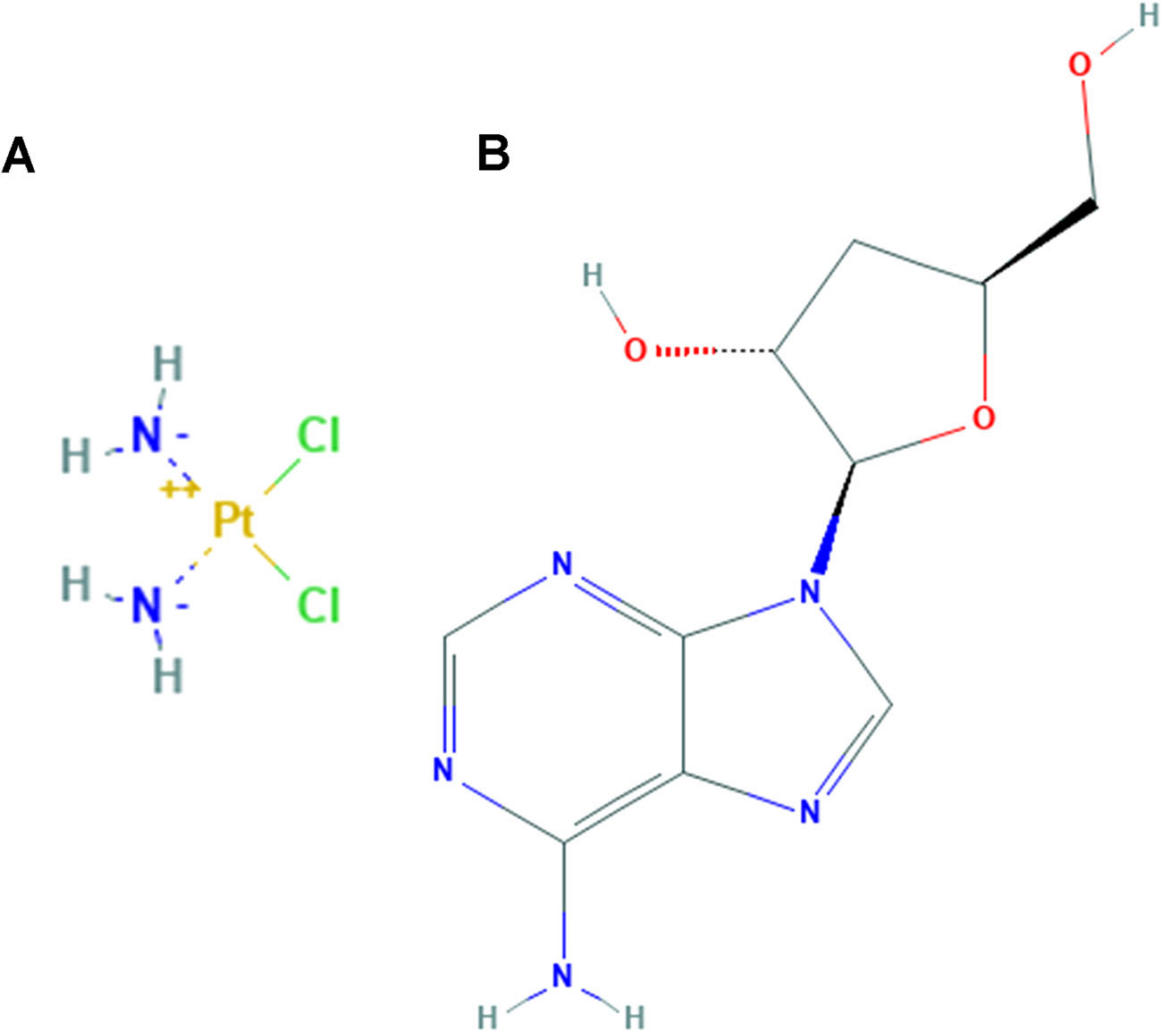
The two-dimensional structure of a drug. Two-dimensional structure of (**A**) cisplatin and (**B**) cordycepin (obtained from PubChem compound http://pubchem.ncbi.nlm.nih.gov/).

In this study, we investigated the effects of combined treatment with cordycepin and cDDP (Fig. 1) on the cell growth and apoptosis of esophageal cancer cell lines and examined the effect of cordycepin on AMPK and AKT signaling. In addition, the combination therapy of cordycepin and cDDP was analyzed in vivo using an esophageal cancer xenograft model (Table 1). In clinical practice, the results of this study may provide an alternative treatment with an improved prognosis for patients with esophageal cancer.

**Table 1:** Summary of CI value and the concentration of separate drugs in combination at 50% Fa.

Drug combination	Fa = 0.5			
	HK	K80	EC109	K70
Cor + cDDP				
CI	0.55126	0.63758	0.64362	0.68663
Cor (μM)	0.85734	0.85423	0.95726	0.95387
cDDP (μM)	1.56725	1.78625	1.89868	1.92457

## Results

### Co-treatment with cordycepin and cDDP synergistically inhibits esophageal cancer cell proliferation

**Fig. 2:**
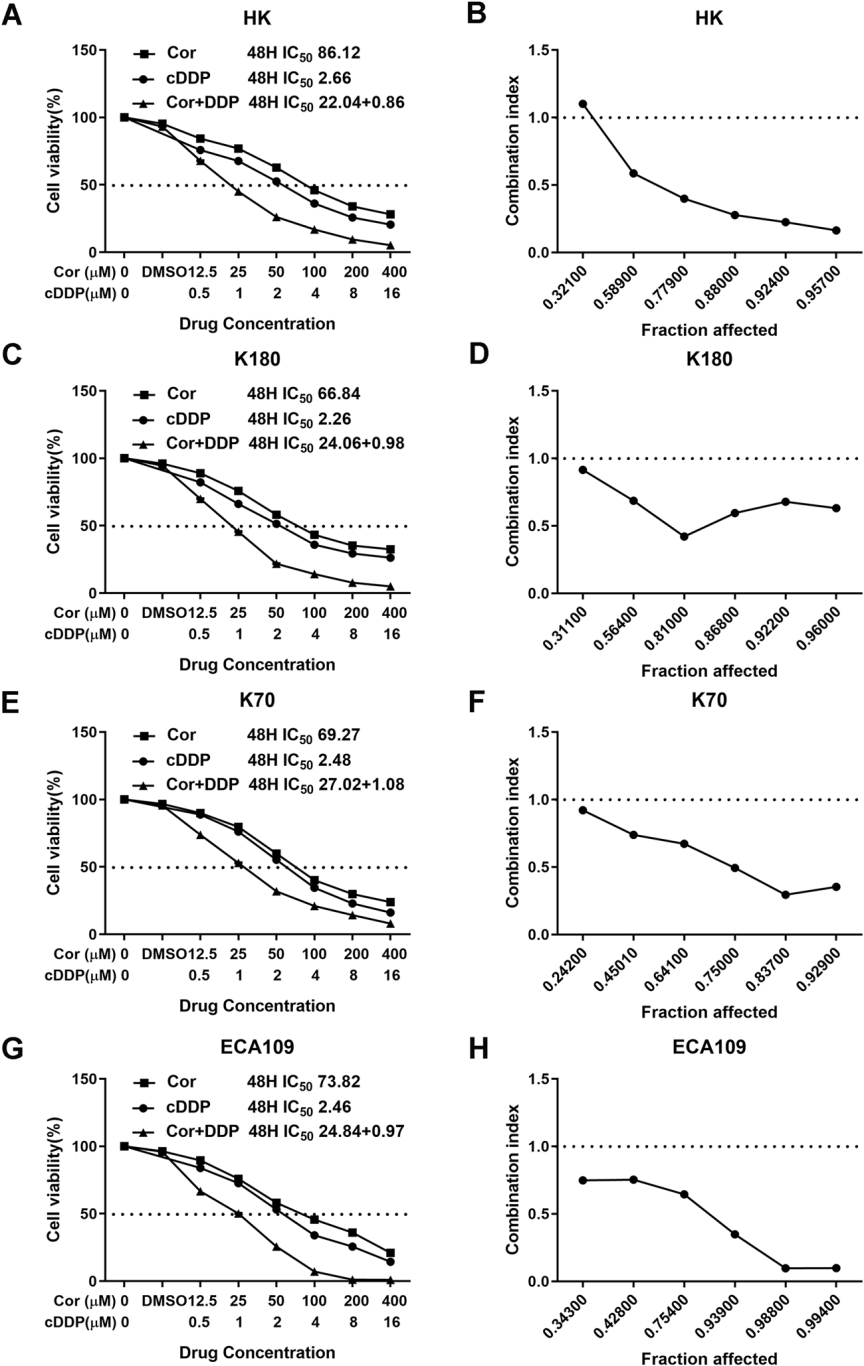
The inhibitory effect of cordycepin, cDDP, and combination therapy on the proliferation of esophageal cancer cells. Cordycepin, cDDP, and combination therapy inhibited the proliferation of esophageal cancer cells as shown by decreased cell viability (**A**, **C**, **E**, **G**). The drug concentration-cell viability curve was generated as a percentage of viable cells. The synergistic effect between cordycepin and cDDP is represented by a Fa-CI diagram (**B**, **D**, **F**, **H**). The above data are from three replicate experiments in quadruplicate wells (mean ± SD). Compared with the control group, *p < 0.05, **p < 0.01 or ***p < 0.001. cDDP cisplatin, Cor cordycepin.

**Fig. 3:**
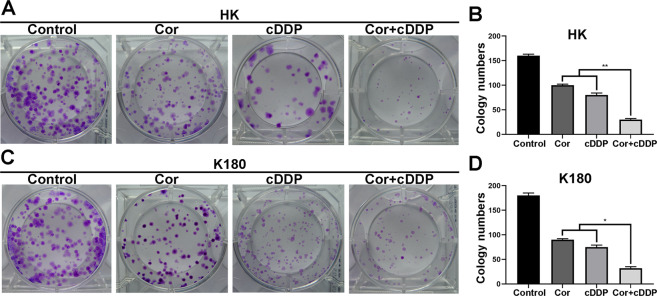
Cordycepin enhances the role of cDDP in esophageal cancer cells. Clonal survival analysis showed the number of colonies of K180 and HK cells after treatment with cordycepin (50 μM), cDDP (2 μM), or the combination of them (25 μM cDDP and 1 μM Cordycepin) (**A**, **C**). Statistical analysis of the number of colonies in HK and K180 cells treated with drugs at each specified concentration (**B**, **D**). Data are expressed as (mean ± SD); *n* = 3; *p < 0.05, relative to each group treated with only one drug.

Cordycepin and cDDP were both shown to inhibit esophageal cancer cell proliferation in a dose-dependent manner. Cordycepin and cDDP were used to treat the esophageal cancer cell lines: HK, K180, K70, and ECA109. After 48 h, the IC_50_ values of these two drugs in the above cells were calculated as follows: 86.12, 66.84, 69.27, and 73.82 μM for cordycepin and 2.66, 2.66, 2.48, and 2.46 μM for cDDP, respectively (Fig. 2). Consistent with the IC_50_ of cordycepin and cDDP, HK, K180, K70 and ECA109 cells were each treated with the specified concentrations of cordycepin and cDDP for 48 h. Compared with the group treated with one drug alone, the combined effect of cordycepin and cDDP on the inhibition of cell proliferation was enhanced (Fig. 2A, C, E, G). In order to further determine the effects of cordycepin and cDDP on esophageal cancer cells, we also performed plate cloning experiments. Subsequently, we also calculated combination index (CI) to determine if cordycepin and cDDP combination therapy had synergistic, additive, or antagonistic effects (Table 1). The results showed that cordycepin and cDDP (CI < 1) had a synergistic effect on esophageal cancer cell proliferation inhibition after 48 h of treatment (Fig. 2B, D, F, H). After treatment with cordycepin (50 μM) and cDDP (2 μM) for 10 days, it was observed that colony formation in HK and K180 esophageal cancer cells was also significantly inhibited (Fig. 3A–D).

### Combination of both cordycepin and cDDP induces cellular apoptosis of esophageal cancer

**Fig. 4:**
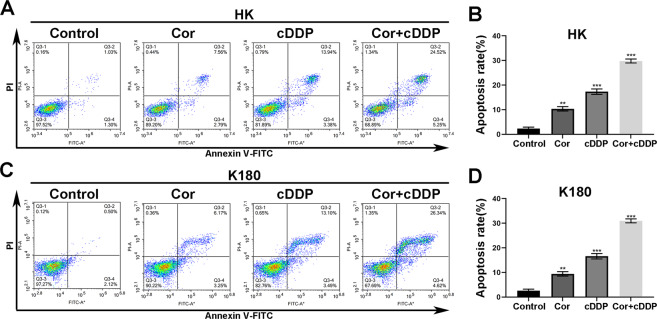
Effect of cordycepin and cDDP alone or in combination on apoptosis of esophageal cancer cells. A representative map shows apoptosis in (**A**) HK or (**C**) K180 cells after 48 h of treatment with either cordycepin (50 μM) or cDDP (2 μM) alone, or cDDP (1 μM) and cordycepin (25 μM) combined. The data represent the number of cells in (**B**) HK and (**D**) K180 cells during cell cycle arrest. All data are shown as the (mean ± SD) of three independent experiments. *** Compared with the control group, *p* < 0.001. cDDP cisplatin, Cor cordycepin [color numbers can be viewed on wileyonlinelibrary.com].

After verifying the antiproliferative effects of cordycepin and cDDP, we further investigated whether cordycepin and cDDP could induce cell apoptosis in esophageal cancer cells. HK and K180 esophageal cancer cells were each treated with specific concentrations of cordycepin and/or cDDP. After 48 h, Annexin V/PI staining assay was performed with flow cytometry to assess the number of apoptotic cells. The obtained results showed that the combined treatment significantly enhanced the apoptosis of HK and K180 esophageal cancer cells after 48 h compared to the control group, as well as using only one drug alone (Fig. 4A–D).

### Co-treatment with cordycepin and cDDP synergistically inhibits esophageal cancer cell migration and invasion

**Fig. 5:**
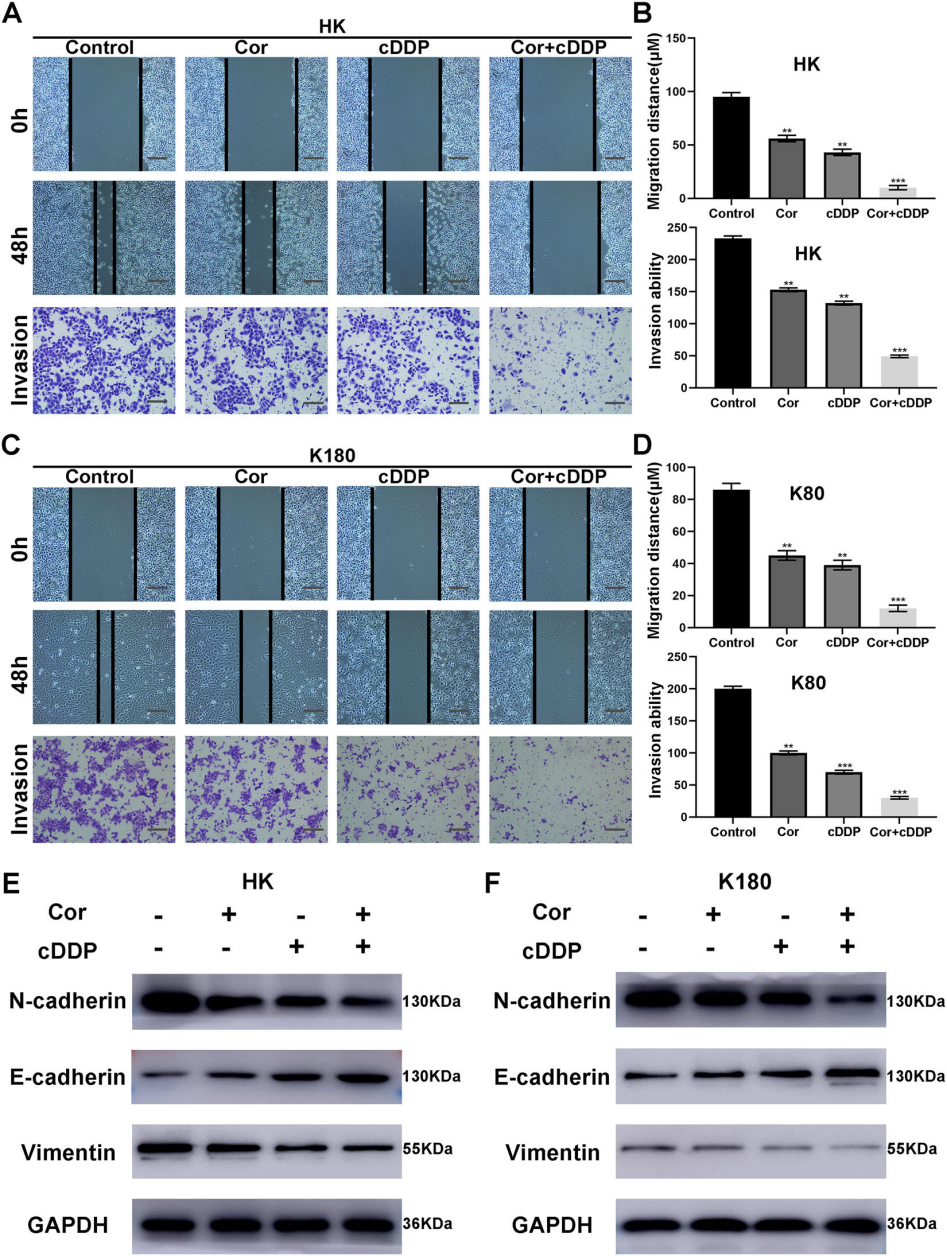
Cordycepin and cDDP inhibit the migration and invasion ability of K180 and HK cells. Representative images of wound healing (**A**, **C**) and perforation determination (**A**, **C**) after 48 h of treatment with either cordycepin (50 μM) or cDDP (2 μM) alone, or cDDP (1 μM) and cordycepin (25 μM) combined. The histogram describes the average migration distance (**B**, **D**) and the number of invasive cells (**B**, **D**), respectively. In addition, the expression of EMT-related proteins was detected (**E**). All data are shown as the (mean ± SD) of three independent experiments. **p < 0.01 or ***p < 0.001 relative to the control group (magnification ×100; scale bar 100 μm). DDP cisplatin, Cor cordycepin. [color numbers can be viewed on wileyonlinelibrary.com].

To identify whether the combination of cordycepin and cDDP affected other biological functions, we further tested the migration and invasion ability of HK (Fig. 5A, B) and K180 (Fig. 5C, D), using a wound healing and transwell assay. The results showed that after 24 h of drug treatment, the number of esophageal cancer cells decreased significantly. Compared with the single-drug treatment, the combined therapy had the smallest migration distance and aggressive cell number. In addition, we tested the expression level of EMT (Epithelial–mesenchymal transition) protein. The results showed that in the combined treatment group of cordycepin and cDDP, the expression of N-cadherin and Vimentin was significantly down-regulated, while the expression of E-cadherin was upregulated compared with the single-drug group (Fig. 5E, F). The above results indicate that cordycepin and cDDP synergistically inhibit the migration of esophageal cancer cells and are associated with EMT.

### Cordycepin enhances the sensitivity of esophageal cancer cells to cDDP by inhibiting the AKT signaling pathway

**Fig. 6:**
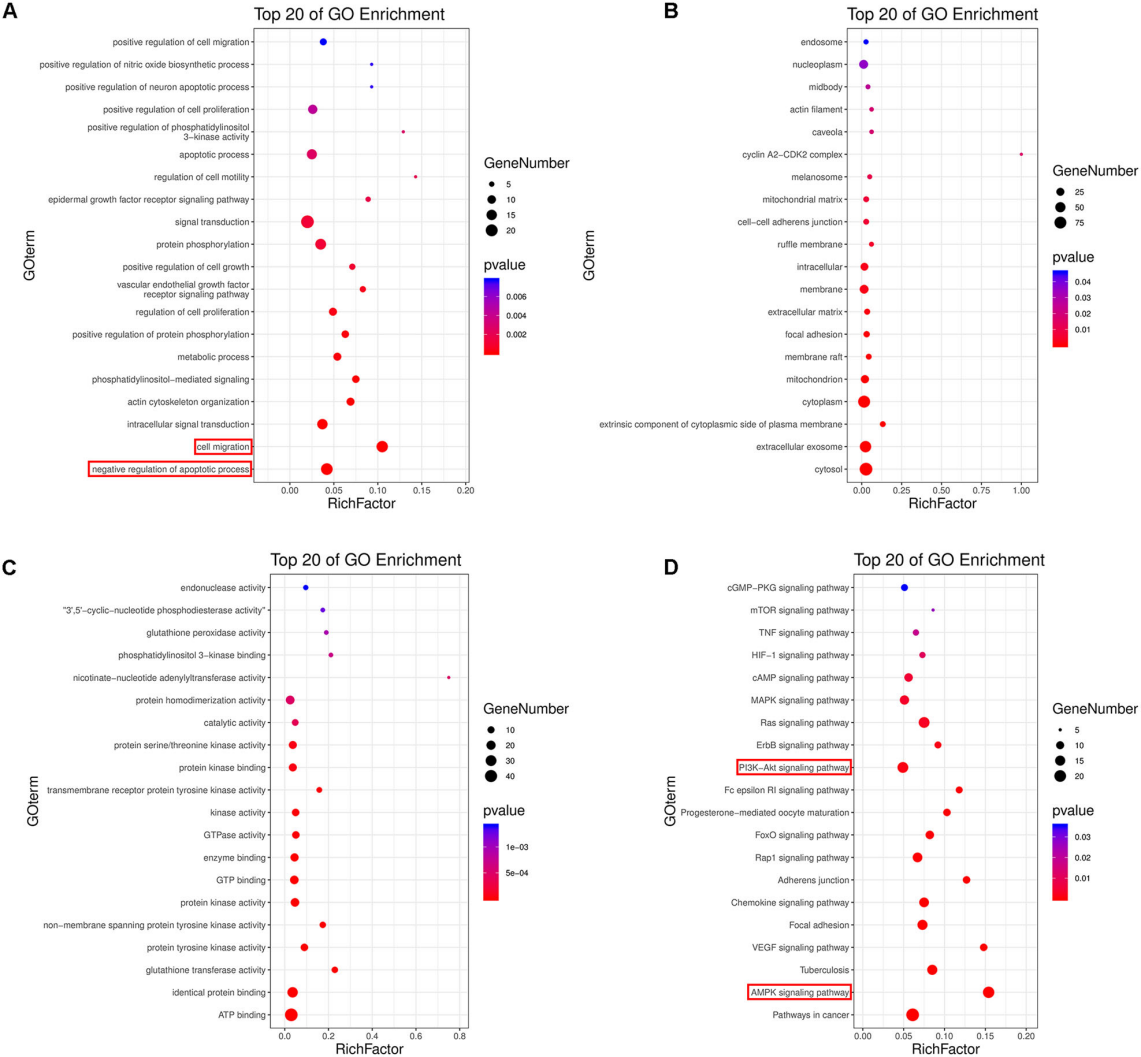
GO and KEGG pathway analysis of cordycepin. The Top 20 signaling pathways of Tan IIA in GO-BP pathway analysis (**A**), GO-CC (**B**), GO-MF (**C**), the Top 20 signaling pathways of cordycepin in KEGG pathway analysis (**D**).

Using Pharma Mapper, we obtained information about the top 300 potential protein targets for cordycepin (Table S1), Cordycepin inhibits lipopolysaccharide (LPS)-induced tumor necrosis factor (TNF)-α production by activating AMP-activated protein kinase (AMPK) signaling. We analyzed the signaling pathways using GO and KEGG analysis. The results showed pathway enrichment for the GO term “cell migration”, “negative regulation of apoptotic process”, “PI3K-AKT signaling pathway”, and “AMPK signaling pathway” (Fig. 6A–D).

### Co-treatment of cordycepin and cDDP synergistically reduce the activity of the PI3K/AKT signaling pathway in esophageal cancer cells

**Fig. 7:**
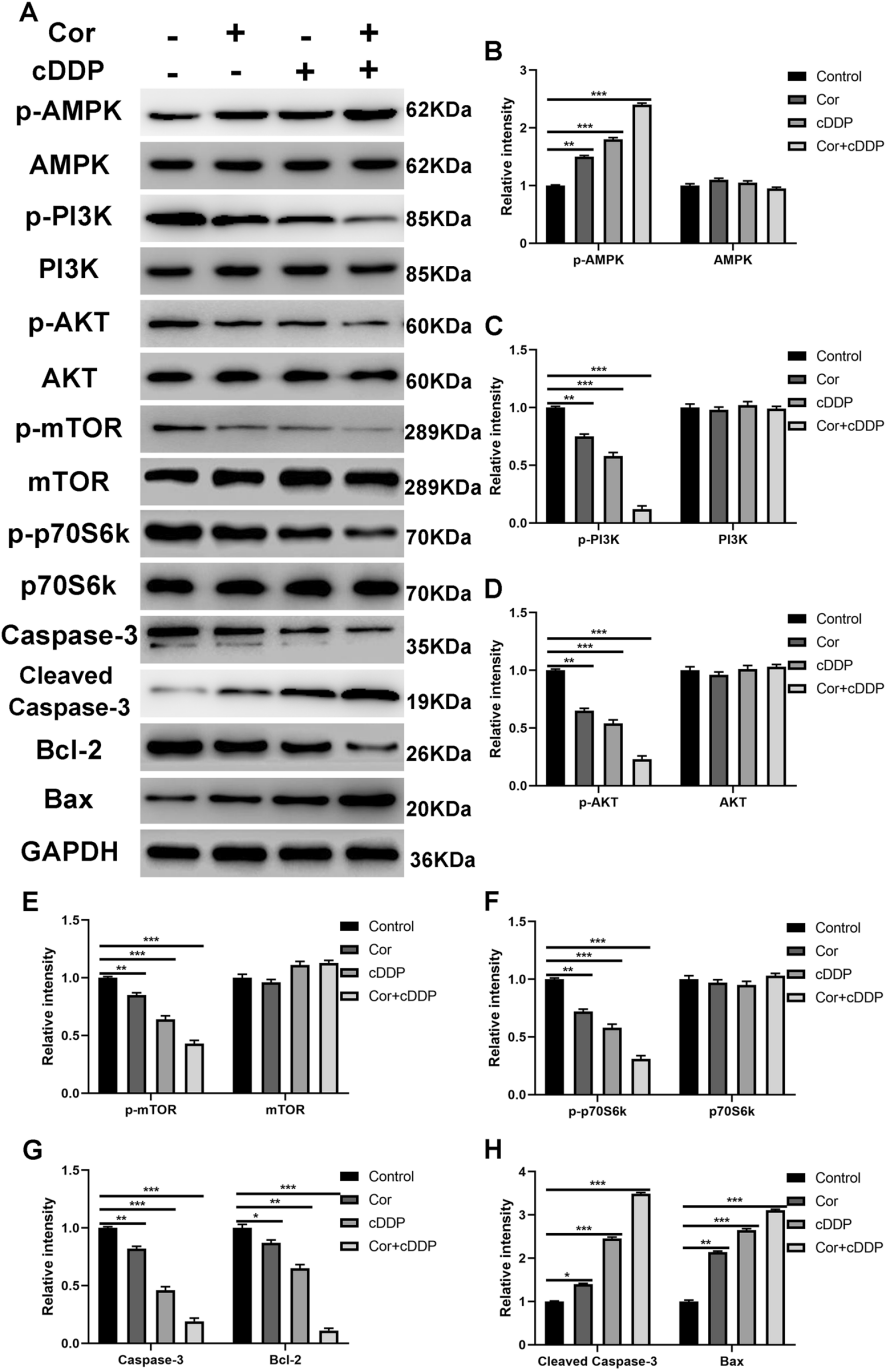
Cordycepin, cDDP, and the combination of the two inhibit the PI3K/AKT signaling pathway in K180 cells. Protein expression levels of p-PI3K, PI3K, p-Akt, Akt, Caspase-3, cleaved Caspase-3, Bcl-2, Bax, and GAPDH in K180 cells treated with either cordycepin (50 μM) or cDDP (2 μM) alone, or cDDP (1 μM) and cordycepin (25 μM) for 48 h (**A**). The histogram describes the relative gray values of related proteins measured using Image J (**B**–**H**). All data are shown as the (mean ± SD) of three independent experiments. **p < 0.01 or ***p < 0.001 compared with the control group. cDDP cisplatin, Cor cordycepin, P13K phosphatidylinositol 3-kinase, GAPDH glyceraldehyde 3-phosphate dehydrogenase.

The GO analysis results indicated that co-treatment of cordycepin and cDDP functions by activating the AMPK signaling pathway. For further verification, we performed western blot analysis on HK and K180 cell lines after monotherapy and combination therapy. We found that the protein expression levels of p-AMPK, cleaved Caspase-3, and Bax were upregulated in cells treated with monotherapy and combination therapy, while the levels of p-PI3K, p-Akt, Caspase-3, and Bcl-2 were down-regulated, and the total Akt, PI3K and GAPDH levels remained unchanged. Importantly, compared with the single-drug treatment group, the efficacy of the drug combination treatment group showed significant differences (cordycepin + cDDP and cordycepin **p < 0.1; cordycepin + cDDP and cDDP, **p < 0.1), as shown in Fig. 7A–H.

### Combined treatment of cordycepin and cDDP synergistically inhibits growth of esophageal cancer cell xenograft tumors

**Fig. 8:**
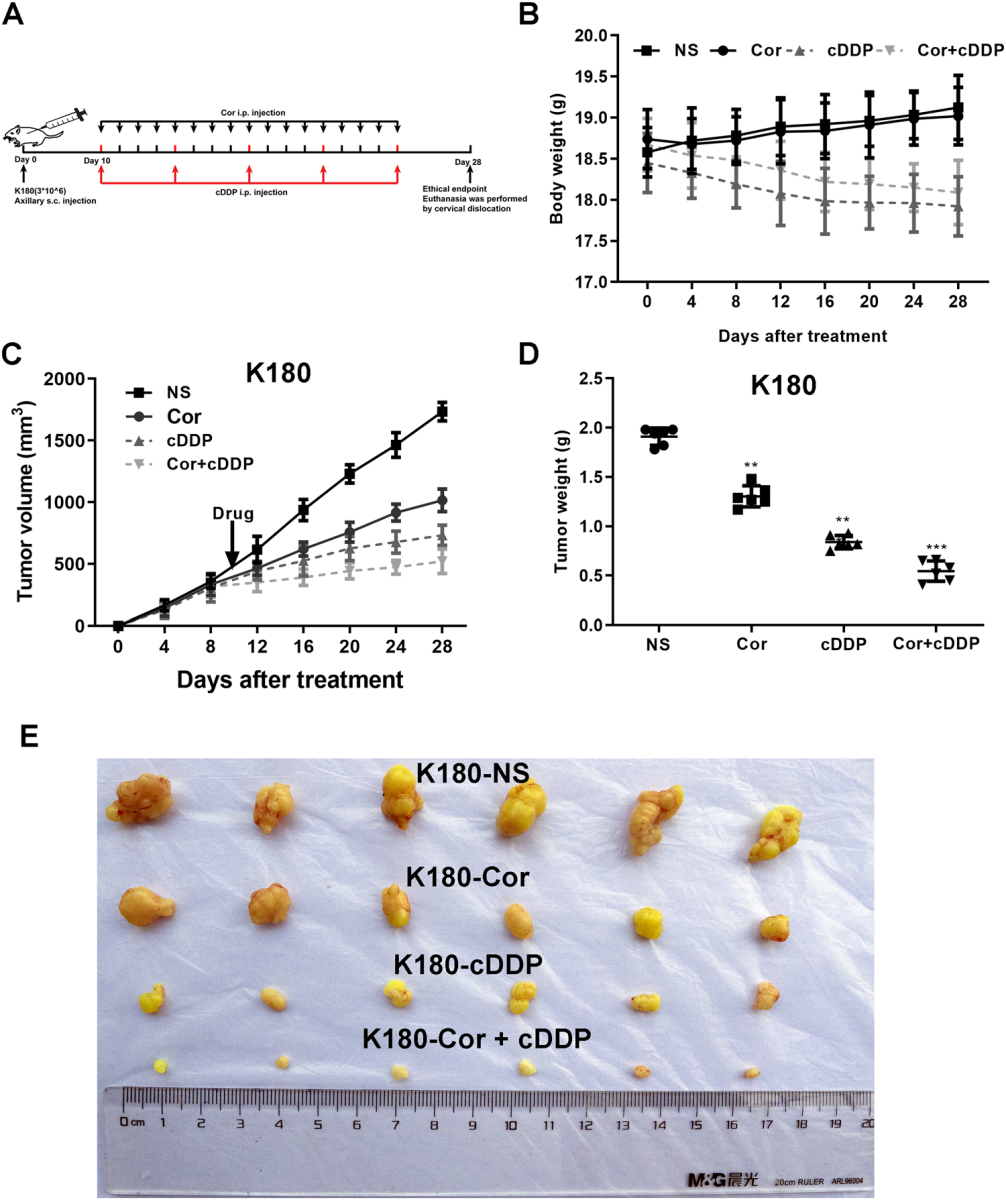
The ability of cordycepin, cDDP, and the combination of the two to inhibit the growth of K180 cells in vivo. K180 cells (3 × 10^6^ cells) were injected subcutaneously into nude mice, and cordycepin and/or cDDP (control group; cordycepin treatment group (75 mg/kg); cDDP treatment group (3 mg/kg); cordycepin (25 mg/kg) and cDDP (1 mg/kg) combined treatment group) were injected intraperitoneally every 4 days after 10 days (**A**). Changes in the body weight of all groups were measured and compared (**B**). From the first day after injection until the end of the study, the tumor volume of all groups was measured every 4 days. The growth curve compares the tumor volume (**C**) and weight (**D**) of all groups, compared with the control group, ***p < 0.001. Tumors derived from K180 cells in six male nude mice are shown (**E**). [Color numbers can be found on wileyonlinelibrary.com].

Next, we studied the in vivo efficacy of combined treatment of cordycepin and cDDP. Nude mice were xenografted with esophageal cancer cells and were randomly divided into four groups. The four groups of nude mice were treated with different drugs (control group; cordycepin treatment group (75 mg/kg); cDDP treatment group (3 mg/kg); cordycepin (25 mg/kg) and cDDP (1 mg/kg) combined treatment group) (Fig. 8A, B). Compared to the control group, the xenograft tumors in the drug treatment groups grew more slowly (Fig. 8C–E). Compared with single-drug therapy, the tumour-suppressive effect of combination therapy was more significant. Therefore, the data suggest that cordycepin can enhance the antitumor effect of cDDP in vivo.

## Discussion

This study has demonstrated for the first time the ability of cordycepin to synergize the anti-cancer effects of cDDP in esophageal cancer cell lines. Based on the current challenges in esophageal cancer treatment, combinational use of cordycepin and cDDP may serve as a possible emerging adjuvant therapy. Due to the clinical success of artemisinin (qinghaosu)^15^ and arsenic (III) oxide (As_2_O_3_)^16^, many researchers have generated great interest in natural extracts. There is an increasing number of researchers who are studying the safety of natural extracts and long-term use, as well as targeting multiple pathways, and an in-depth exploration of the molecular mechanism of its activity^17–20^.

Previous studies have provided evidence of increased efficacy of natural drugs in combination with other therapeutic agents, such as Tan IIA^21^, curcumin^22^, fucoxanthin^23^, and matrine^24^. However, no one has studied the effect and mechanism of combination therapy of cordycepin and cDDP on the development of esophageal cancer. Through this study, we observed that cordycepin reduced the ability of esophageal cancer cells exposed to cDDP to proliferate, invade and metastasize, and induce cellular apoptosis. Its mechanism of action is produced by mediating AMPK activation and inhibiting the AKT signaling pathway. Based on its known anti-cancer effect, the ability of cordycepin and cDDP to synergistically treat tumor cells is surprising. Therefore, cordycepin may be an effective anti-cancer drug in preclinical models of esophageal cancer.

The IC_50_ value analysis of the Cell Counting Kit-8 proved that cordycepin and cDDP can inhibit the proliferation of HK, K180, K70, and ECA109 esophageal cancer cells in a dose- and time-dependent manner. As cordycepin alone has a relatively weak inhibitory effect on the proliferation of esophageal cancer cell lines and has a synergistic effect when used in combination with cDDP, we speculate that cordycepin plays a synergistic role with cDDP in the treatment of esophageal cancer. Many studies have demonstrated that cordycepin results in an increased apoptosis rate in oral squamous carcinoma cells^25^, bladder cancer T24 cells^26^, and GBC-SD gallbladder cancer cells^27^. In addition, we found that cordycepin can effectively sensitize cisplatin-resistant bladder cancer cells to cDDP^28^. Our results also show that both cordycepin and cDDP produce the same knots in HK and K180 cells. Compared with drug treatment alone, the combination of the two drugs can significantly increase the apoptosis rate of esophageal cancer cells. Taken together, these results indicate that the combination of cordycepin and cDDP has a synergistic effect on inhibiting apoptosis. Other studies have shown that cordycepin can inhibit the invasion and metastasis of cancer cells^29–32^. Meanwhile, through our research, we found that cordycepin and cDDP can synergistically inhibit the ability of esophageal cancer cells to invade and metastasize and associate with EMT. In addition, this study also explained the synergistic mechanism of cordycepin with cDDP on esophageal cancer cells. Different drugs were used to treat esophageal cancer cells separately and in combination. Western blot analysis revealed that compared with the group treated with one drug alone, the changes in related protein expression levels were more significant after combined treatment with cordycepin and cDDP. Significantly, the expression levels of Bax and cleaved Caspase-3 were upregulated, while the expression levels of p-PI3K, p-Akt, Caspase-3, and Bcl-2 were down-regulated. Therefore, cordycepin enhances the sensitivity of cDDP in esophageal cancer cells in part by activating AMPK, while blocking the AKT signaling pathway.

To verify this effect in vivo, we used an esophageal cancer cell xenografted animal model to compare tumor growth size. It was found that the combined treatment significantly reduced tumor growth in animals.

Studies have found that cordycepin promotes the anti-tumor efficacy of certain chemotherapy treatments, including the therapeutic effects of gemcitabine and 5-fluorouracil on GBC-SD cells^27^. It is possible that this is due to cordycepin-induced AMPK activation during combined use of cordycepin and cDDP in esophageal cancer cells. In addition, other studies have found that the combined therapy of cDDP and cordycepin exerts apoptotic effects in human OC3 oral cancer cell lines by activating the JNK/caspase-7/PARP signaling pathway^33^. Therefore, it can be speculated that cordycepin may enhance the anti-cancer ability of cDDP in esophageal cancer by mediating AMPK activation and inhibiting the AKT signaling pathway.

**Fig. 9:**
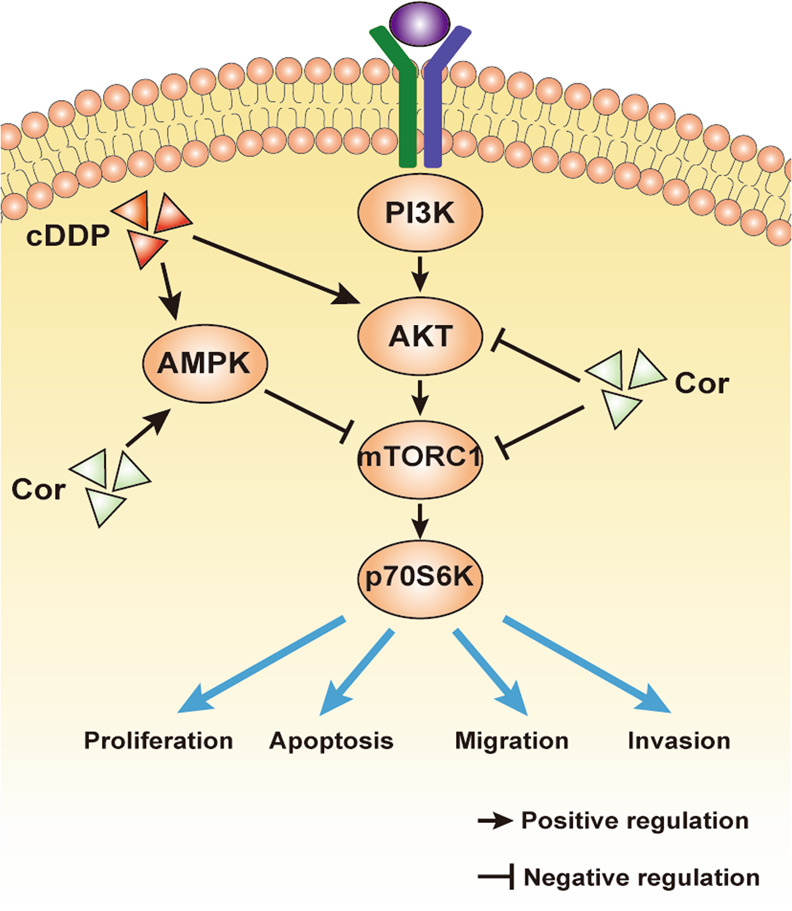
Drug action diagram. Schematic diagram depicting a proposed model for the role and a major mechanism of co-treatment of cordycepin and cDDP inhibiting esophageal cancer cells.

In conclusion, to further demonstrate the possible mechanism of action after cordycepin and cDDP are combined, we applied an antagonist antibody evaluation to study the possible molecular mechanisms for designing the AMPK and AKT pathways. After our research, we proposed a new model, whereby the synergistic effect of combined treatment with cordycepin and cDDP is mainly achieved by upregulation of the AMPK and downregulation of the AKT signaling pathway in esophageal cancer cells (Fig. 9). Experiments in vivo and in vitro have demonstrated the synergy of these two drugs together. Therefore, the combined application of cordycepin and cDDP may become a new therapy to improve the efficacy of treatment on cDDP-resistant esophageal cancer patients.

## Materials and methods

### Reagents and cell culture

Reagents, including cordycepin and cDDP, were purchased from Sigma (St. Louis, Missouri). Immediately before use, cordycepin was dissolved in dimethyl sulfoxide to make a 10 mM stock solution and cDDP was dissolved in physiological saline to make a 10 mM stock solution. Both of the stock solutions were stored at −20 °C. HK, K180, K70, and ECA109 esophageal cancer cell lines were obtained from the State Key Laboratory of Oncology in South China. The cell lines were all cultured in DMEM (Gibco, Carlsbad, CA, USA) supplemented with 10% fetal bovine serum (Invitrogen Corp, Carlsbad, CA, USA), penicillin (100 U/ml) and streptomycin (100 U/ml, Gino, Hangzhou, Zhejiang, China) in a humidified incubator at 37 °C, 5% CO_2_.

### Cell viability assay

HK, K180, K70, and ECA109 esophageal cancer cells (8.0 × 10^3^ cells per well) were seeded into 96-well plates, 200 μl per well, and cultured overnight in the incubator. The cells were cultured separately and simultaneously with different concentrations of cordycepin and cDDP. After 24, 48, or 72 h of treatment, cells were incubated with 10 μL of CCK-8 (Dojindo Laboratories, 119 Kuma- moto, Japan) solution for an additional 90 min. Finally, the optical density was measured using a microplate reader (Thermo Scientific, Rockford, IL, USA) at 450 nm. The proliferation inhibition rate was calculated as follows: proliferation inhibition rate = (1−experimental group/control group) x 100%. SPSS 20.0 software was used to calculate the 50% inhibitory concentration (IC_50_) value by non-linear regression analysis.

### Colony formation assay

The esophageal cancer cells were spread in a 6-well plate. After 24 h of culture, the original medium was replaced with a new medium containing a specified concentration of cordycepin and/or cDDP. Then the cells were cultured for another 10 days. After 10 days, the cells were fixed with a paraformaldehyde solution and stained with crystal violet. Under a microscope, colonies >50 cells were observed and counted at ×4 magnification.

### Transwell and wound healing assay

Transwell filters (pore size = 8 µm, polycarbonate = 6.5 mm, Corning, NY, USA) with a thin Matrigel matrix (BD Biosciences, Bedford, Massachusetts) were used. Esophageal cancer cells (cell number: 3 × 10^4^) in the logarithmic phase were resuspended in 500 μL of serum-free medium treated with different drugs. After the cells were resuspended, the resuspension was spread in the upper compartment of the transwell and 800 μL of complete medium containing 10% FBS was added to the lower compartment. The 24-well plate was placed in a humidified incubator at 37 °C, 5% CO_2_. After 24 h of incubation, the plate was removed, fixed with 4% paraformaldehyde at room temperature for 30 min, and stained with crystal violet (0.5%). After the cells on the upper surface of the filter were removed by wiping with a cotton swab, the number of stained cells on the lower surface was counted by a microscope in three random fields per filter (magnification, ×100). Each transwell filter counts five fields. For wound healing assays, log-phase esophageal cancer cells were seeded into 6-well plates. After the cells grew to 80% confluency, the adhered cells were scraped in a straight line with a 200 µL pipette tip, removed, and washed three times with phosphate-buffered saline (PBS). Fresh serum-free medium and serum-free medium containing drugs were added to the 6-well plate and the cells were repaired for scratches for 24 h. Images of the scratched positions at 0 and 24 h were recorded under a microscope (magnification, ×10). Then, the distance of the cell movement was measured using Adobe Photoshop CS6 software.

### Apoptosis assay

Apoptosis was assessed using Annexin V-FITC Apoptosis Detection Kit (USA, BD) according to the manufacturer’s protocol. Cells in the logarithmic phase were transferred to a 6-well plate (2 × 10^5^/well). After the specific treatment of each group, FACSCalibur flow cytometer (BD, USA) was used to collect at least 10,000 cells. Of cells that were used for fluorescence-activated cell sorting, the proportion of apoptotic cells and viable cells in each group was determined separately.

### Mouse xenograft model

Four-week-old female nude mice were randomly grouped for animal experiments. An equal amount of K180 cells (3 × 10^6^ cells) were resuspended in 100 mL of physiological saline and injected subcutaneously into the unilateral axilla of nude mice (BALB/c-nu). The formula for calculating tumor volume is as follows: *V* = 0.5 *ab*2 (*a*, the longest tumor axis; *b*, the shortest tumor axis). When the tumor volume reached 300 mm^3^, mice were given equal volumes of cordycepin (75 mg/kg), DDP (3 mg/kg), cordycepin (37.5 mg/kg), and cDDP (1.5 mg/kg) or Vehicle (saline) was injected intraperitoneally twice a week. At the end of the study, we excluded the dead mice, euthanized them by cervical dislocation, and removed the tumors. Finally, six mice were randomly selected for each group. All studies were reviewed and approved by the SYSUCC Institutional Animal Care and Use Committee. All animal experiments were performed in accordance with the Guidelines for the Care and Use of Laboratory Animals (NIH publications Nos. 80–23, revised 1996).

### Western blot

Log-stage esophageal cancer cells were treated with different drugs. After 48 h, harvested esophageal cancer cells were lysed with lysis buffer. Cell lysate was incubated on ice for 30 minutes and then centrifuged at 4 °C (12,000 × *g*, 10 min). The supernatant was collected and protein content was measured using a BCA protein assay kit (Beyotime, Jiangsu, China). Selected protein extracts were separated using 10% sodium lauryl sulfate-polyacrylamide gel electrophoresis and then transferred to a PVDF membrane (0.22 μm, Millipore, MA, USA). After blocking with 5% skim milk for 1 h, primary antibody (p-Akt CST #4060 S 1: 1000, Akt CST #4691 S 1: 1000, p-PI3K CST #17366S 1: 1000, PI3K CST #4249S 1: 1000, Caspase-3 CST #9662S 1: 1000, cleaved Caspase-3 CST #9661S 1: 1000, Bcl-2 CST #15071S 1: 1000, Bax CST #5023S 1: 1000, and GAPDH CST #5174S 1: 8000) was incubated overnight at 4 °C. All primary antibodies were purchased from Cell Signaling Technology (Denver, Massachusetts, USA). After washing three times with Tris-buffered saline (containing 0.1% Tween-20 (TBST)), the membrane was incubated with secondary antibody (Cell Signaling Technology, Danvers, MA, USA) at room temperature for 1 h, and washed again three times with TBST. The membrane’s immunoreactivity was tested by Bio-Rad-Image-Lab and an electrochemiluminescence system (Thermo Fisher Scientific, MA, USA). The optical density of protein bands was measured using Image J (NIH image software) and its related controls were standardized.

### Statistical analysis

After all experiments were repeated three times, the data obtained were expressed as mean ± SD. Data were analyzed using GraphPad Prism 6.02 software (San Diego, California, USA), but IC_50_ values calculated using SPSS 20.0 software were not included. Differences between groups were analyzed using Student’s *t*-test. A *p*-value of 0.05 or less was considered significant.

## Supplementary information

Supplement table1

## Data Availability

All data generated or analyzed during this study are included in this published article.
