# Supplementary material for: Cordycepin enhances the chemosensitivity of esophageal cancer cells to cisplatin by inducing the activation of AMPK and suppressing the AKT signaling pathway
Source: Cell Death Dis. 2020 Oct 16;11(10):866. doi: 10.1038/s41419-020-03079-4 (PMC7567864; doi:10.1038/s41419-020-03079-4)
Supplement: Supplementary file 1 — Supplement table1 [file 41419_2020_3079_MOESM1_ESM.docx]

Supplement table1 Potential targets of cordycepin by PharmaMapper

| **PDB ID** | **Target name** | **Num Feature** | **Fit** | **Norm Fit** | **Z score** |
| --- | --- | --- | --- | --- | --- |
| 2itx | EGFR | 3 | 2.819 | 0.9398 | 0.992558 |
| 1xn0 | PDE4B | 3 | 2.757 | 0.9189 | 0.854357 |
| 3fzh | HSPA8 | 3 | 2.703 | 0.9011 | 0.799521 |
| 1ogs | GLCM | 3 | 2.624 | 0.8746 | 0.542264 |
| 1cb0 | MTAP | 3 | 2.493 | 0.8309 | 0.249159 |
| 3d2e | SSE1 | 6 | 4.902 | 0.8171 | 4.27356 |
| 1bx4 | ADK | 8 | 6.338 | 0.7922 | 3.88133 |
| 2o63 | PIM1 | 3 | 2.317 | 0.7722 | 0.285434 |
| 2cgx | CHK1 | 3 | 2.202 | 0.7339 | -0.0578276 |
| 1a7a | SAHH | 5 | 3.518 | 0.7036 | 2.05871 |
| 1xlw | CHLE | 3 | 2.096 | 0.6985 | -0.13647 |
| 1v45 | PNPH | 4 | 2.693 | 0.6732 | 0.720679 |
| 1e1v | CDK2 | 4 | 2.666 | 0.6666 | 0.396043 |
| 1fa9 | PYGL | 4 | 2.646 | 0.6614 | 0.497687 |
| 1dmw | PH4H | 5 | 3.27 | 0.6539 | 1.49229 |
| 1k59 | ANGI | 4 | 2.586 | 0.6465 | -0.0520928 |
| 3h0w | AMD1 | 4 | 2.544 | 0.636 | 0.353736 |
| 1wuu | GALK1 | 5 | 3.175 | 0.6351 | 1.04731 |
| 1i72 | DCAM | 4 | 2.513 | 0.6282 | 0.469952 |
| 1t7v | ZA2G | 4 | 2.476 | 0.6191 | 0.349774 |
| 1csb | CATB | 4 | 2.41 | 0.6024 | -0.102599 |
| 1m4u | BMP7 | 4 | 2.406 | 0.6015 | -0.00402313 |
| 1p5z | DCK | 5 | 2.994 | 0.5988 | 1.19419 |
| 1isg | BST1 | 6 | 3.575 | 0.5959 | 2.12633 |
| 1ksw | SRC | 5 | 2.908 | 0.5817 | 0.899162 |
| 3cqw | AKT1 | 5 | 2.902 | 0.5803 | 0.964547 |
| 1jqh | IGF1R | 5 | 2.896 | 0.5792 | 0.788984 |
| 1wlj | ISG20 | 5 | 2.884 | 0.5767 | 0.327873 |
| 1hkc | HXK1 | 5 | 2.88 | 0.576 | 0.709126 |
| 2vwv | EPHB4 | 5 | 2.859 | 0.5718 | 0.598075 |
| 1mfu | AMY1A | 4 | 2.253 | 0.5633 | -0.499029 |
| 1u4d | ACK1 | 5 | 2.811 | 0.5621 | 0.552669 |
| 1awb | IMPA1 | 6 | 3.318 | 0.553 | 0.0160506 |
| 1pyx | GSK3B | 5 | 2.764 | 0.5528 | 0.65389 |
| 1pkg | KIT | 7 | 3.861 | 0.5516 | 2.59226 |
| 1uyg | HS90A | 8 | 4.395 | 0.5494 | 2.09661 |
| 1mqb | EPHA2 | 7 | 3.83 | 0.5471 | 2.53439 |
| 1t2f | LDHB | 5 | 2.733 | 0.5466 | -0.0749908 |
| 1g8i | NCS1 | 5 | 2.694 | 0.5388 | 0.609259 |
| 1fe0 | ATOX1 | 5 | 2.661 | 0.5321 | 0.592312 |
| 1yk7 | CATK | 5 | 2.629 | 0.5258 | 0.271926 |
| 1kpe | HINT1 | 8 | 4.197 | 0.5247 | 3.04095 |
| 1g4o | CAH2 | 5 | 2.6 | 0.5199 | -0.156346 |
| 1jap | MMP8 | 5 | 2.598 | 0.5196 | 0.143875 |
| 1qdd | REG1A | 5 | 2.594 | 0.5188 | 0.288551 |
| 1wma | CBR1 | 5 | 2.577 | 0.5155 | 0.0692616 |
| 2uvy | PRKACA | 5 | 2.577 | 0.5155 | 0.32797 |
| 1rem | LYZ | 6 | 3.083 | 0.5139 | 1.35265 |
| 1qpc | LCK | 9 | 4.613 | 0.5126 | 1.99265 |
| 1oix | RB11A | 6 | 3.075 | 0.5124 | 1.04063 |
| 1sc8 | UROK | 5 | 2.54 | 0.508 | 0.387465 |
| 1zdz | SRM | 7 | 3.523 | 0.5033 | 1.79624 |
| 1r7t | ABO | 6 | 3 | 0.5001 | 0.775408 |
| 1jst | CCNA2 | 6 | 2.975 | 0.4958 | 0.954335 |
| 1n6i | RAB5A | 6 | 2.974 | 0.4957 | 0.975836 |
| 1elv | C1S | 6 | 2.958 | 0.493 | 0.647083 |
| 1jwh | CSNK2A1 | 5 | 2.461 | 0.4923 | -0.019807 |
| 1mlw | TPH1 | 7 | 3.435 | 0.4907 | 1.10472 |
| 2rnf | RNASE4 | 5 | 2.418 | 0.4837 | -0.357052 |
| 3cpu | AMYP | 5 | 2.415 | 0.4829 | -0.135438 |
| 1ptw | PDE4D | 8 | 3.84 | 0.48 | 1.25043 |
| 1v84 | B3GA1 | 6 | 2.869 | 0.4781 | 0.756726 |
| 1ds6 | RAC2 | 5 | 2.339 | 0.4679 | -0.309861 |
| 1t9s | PDE5A | 7 | 3.267 | 0.4667 | 1.15836 |
| 1hlc | LEG2 | 6 | 2.786 | 0.4643 | 0.442608 |
| 1bmm | THRB | 6 | 2.782 | 0.4636 | 0.537258 |
| 1kmq | RHOA | 5 | 2.315 | 0.4631 | -0.220823 |
| 2pe2 | PDPK1 | 5 | 2.315 | 0.463 | -0.742604 |
| 1fo2 | MAN1B1 | 5 | 2.314 | 0.4627 | -0.233674 |
| 1w0h | ERI1 | 10 | 4.604 | 0.4604 | 1.95373 |
| 1mx9 | EST1 | 5 | 2.298 | 0.4595 | -0.371329 |
| 1o6l | AKT2 | 7 | 3.194 | 0.4563 | 1.39206 |
| 1mq0 | CDD | 5 | 2.276 | 0.4553 | -0.761587 |
| 1dyt | RNASE3 | 6 | 2.717 | 0.4528 | -0.0456258 |
| 1c9y | OTC | 5 | 2.244 | 0.4488 | -0.963635 |
| 1d3h | PYRD | 6 | 2.669 | 0.4448 | -0.117536 |
| 1mfv | AMY1A | 6 | 2.652 | 0.4419 | 0.281163 |
| 2qcf | PYR5 | 6 | 2.65 | 0.4417 | 0.184801 |
| 1nw3 | DOT1L | 10 | 4.41 | 0.441 | 2.97548 |
| 1nhx | PCK1 | 6 | 2.62 | 0.4367 | -0.549374 |
| 1uej | UCK2 | 6 | 2.614 | 0.4357 | -0.0480517 |
| 1jkl | DAPK1 | 11 | 4.781 | 0.4347 | 2.27967 |
| 1pq3 | ARGI2 | 7 | 3.042 | 0.4346 | 0.961343 |
| 2nsi | NOS2 | 6 | 2.568 | 0.4281 | 0.179912 |
| 1hy7 | MMP3 | 6 | 2.561 | 0.4268 | -0.803587 |
| 1nf7 | IMDH2 | 6 | 2.561 | 0.4268 | 0.0285545 |
| 1rm8 | MMP16 | 8 | 3.41 | 0.4262 | 1.1752 |
| 1bj4 | GLYC | 6 | 2.553 | 0.4256 | -0.034132 |
| 1hy3 | ST1E1 | 8 | 3.398 | 0.4248 | 1.18042 |
| 1kv3 | TGM2 | 5 | 2.123 | 0.4246 | -0.272618 |
| 1ore | APRT | 7 | 2.968 | 0.4239 | 0.348209 |
| 1iz2 | A1AT | 6 | 2.543 | 0.4238 | -0.229784 |
| 2cm8 | PTN1_HUMAN | 5 | 2.108 | 0.4216 | -0.308577 |
| 1m4b | IL2 | 6 | 2.521 | 0.4202 | -0.117261 |
| 1mp8 | FAK1 | 7 | 2.919 | 0.417 | 0.0168023 |
| 1p4r | PUR9 | 9 | 3.752 | 0.4169 | 1.36396 |
| 1x98 | ALDR | 6 | 2.499 | 0.4166 | -0.715997 |
| 1ne7 | GNPDA1 | 7 | 2.895 | 0.4135 | 0.459726 |
| 1r6t | WARS1 | 11 | 4.548 | 0.4134 | 2.75393 |
| 1iri | G6PI | 7 | 2.893 | 0.4134 | -0.211315 |
| 1ny3 | MAPKAPK2 | 6 | 2.475 | 0.4125 | -0.387831 |
| 1g1r | LYAM3 | 6 | 2.473 | 0.4122 | 0.264912 |
| 2c30 | PAK6 | 6 | 2.456 | 0.4093 | -0.676376 |
| 1z83 | AK1 | 11 | 4.471 | 0.4064 | 3.13851 |
| 1u4l | CCL5 | 6 | 2.427 | 0.4045 | -0.436813 |
| 1tx4 | ARHGAP1 | 6 | 2.426 | 0.4043 | -0.154255 |
| 1so2 | PDE3B | 5 | 2.004 | 0.4008 | -1.48904 |
| 1sgx | TGM3 | 6 | 2.404 | 0.4007 | -0.525412 |
| 1hmp | HPRT1 | 9 | 3.593 | 0.3993 | 1.0051 |
| 1jnk | MK10 | 12 | 4.773 | 0.3978 | 2.47941 |
| 1tw6 | BIRC7 | 6 | 2.387 | 0.3978 | -0.00462367 |
| 4ald | ALDOA | 6 | 2.36 | 0.3934 | -0.877674 |
| 1z57 | CLK1 | 7 | 2.752 | 0.3932 | -0.00167036 |
| 1lf5 | RASH | 8 | 3.143 | 0.3928 | 0.669421 |
| 1u59 | ZAP70 | 7 | 2.739 | 0.3913 | -0.229593 |
| 1w2c | ITPKA | 9 | 3.501 | 0.389 | 1.6865 |
| 1kms | DYR | 7 | 2.711 | 0.3873 | -0.0271121 |
| 1fkg | FKB1A | 7 | 2.709 | 0.3871 | -0.239901 |
| 1kjr | LEG3 | 7 | 2.701 | 0.3859 | 0.599772 |
| 1x6v | PAPSS1 | 7 | 2.7 | 0.3857 | -0.447953 |
| 1mq4 | STK6 | 10 | 3.853 | 0.3853 | 1.15094 |
| 1qcf | HCK | 7 | 2.687 | 0.3838 | -0.486186 |
| 1nmx | KTHY | 7 | 2.683 | 0.3833 | -0.354642 |
| 1jcn | IMDH1 | 7 | 2.663 | 0.3804 | -0.383595 |
| 1xbt | TK1 | 7 | 2.663 | 0.3804 | 0.384113 |
| 1q1z | SULT2B1 | 10 | 3.763 | 0.3763 | 0.180506 |
| 1ls6 | ST1A1 | 12 | 4.498 | 0.3748 | 1.66652 |
| 1xts | Rheb | 7 | 2.578 | 0.3683 | 0.0533973 |
| 1lg1 | CHIT1 | 8 | 2.937 | 0.3671 | 0.503337 |
| 1liw | KPYR | 8 | 2.917 | 0.3647 | -0.0852013 |
| 1ms6 | CATS | 7 | 2.539 | 0.3627 | -0.3984 |
| 1qh5 | HAGH | 7 | 2.536 | 0.3623 | -0.496128 |
| 1a4r | CDC42 | 8 | 2.894 | 0.3618 | -0.102127 |
| 2b8v | BACE1 | 8 | 2.893 | 0.3616 | 0.77412 |
| 2ble | GMPR1 | 10 | 3.612 | 0.3612 | 0.850741 |
| 1nb9 | RFK | 11 | 3.955 | 0.3596 | 1.27014 |
| 1i1n | PIMT | 8 | 2.87 | 0.3587 | 0.905393 |
| 1lzj | ABO | 6 | 2.147 | 0.3578 | -0.816876 |
| 1k9j | CLEC4M | 8 | 2.861 | 0.3576 | 0.920375 |
| 1gkd | MMP9 | 7 | 2.503 | 0.3576 | -0.61813 |
| 1g55 | TRDMT1 | 10 | 3.552 | 0.3552 | 1.32612 |
| 1men | PUR2 | 8 | 2.829 | 0.3537 | -0.199487 |
| 1hrn | RENI | 8 | 2.829 | 0.3536 | 0.423423 |
| 1o7a | HEXB | 8 | 2.816 | 0.352 | -0.302372 |
| 1z6y | ARL5A | 9 | 3.146 | 0.3496 | 0.419658 |
| 1iyh | PTGD2 | 8 | 2.793 | 0.3492 | 0.039926 |
| 1ljr | GSTT2 | 7 | 2.436 | 0.348 | -0.727559 |
| 1efh | SULT2A1 | 11 | 3.82 | 0.3473 | 0.982561 |
| 1p5j | SDS | 7 | 2.428 | 0.3469 | -0.911331 |
| 2fs9 | TRYB2 | 8 | 2.771 | 0.3464 | 0.439284 |
| 1jj7 | TAP1 | 11 | 3.793 | 0.3448 | 0.625541 |
| 2b7a | JAK2 | 7 | 2.404 | 0.3435 | -0.956957 |
| 2aax | MCR | 6 | 2.061 | 0.3435 | -0.72801 |
| 1eem | GSTO1 | 8 | 2.737 | 0.3422 | -0.106117 |
| 1jqd | HNMT | 10 | 3.417 | 0.3417 | 0.769405 |
| 2aeb | ARGI1 | 7 | 2.392 | 0.3417 | -0.583079 |
| 1wms | RAB9A | 9 | 3.032 | 0.3369 | 0.366029 |
| 1qzu | PPCDC | 7 | 2.358 | 0.3368 | -0.514718 |
| 1i44 | INSR | 8 | 2.693 | 0.3366 | -0.71186 |
| 1yq7 | FPPS | 8 | 2.69 | 0.3362 | -0.724509 |
| 1m2z | GCR | 8 | 2.687 | 0.3359 | 0.036944 |
| 1z6x | ARF4 | 8 | 2.675 | 0.3343 | -0.393176 |
| 1upr | PLEKHA4 | 7 | 2.329 | 0.3327 | -1.13375 |
| 1ekv | BCAT2 | 8 | 2.653 | 0.3317 | -0.545098 |
| 1f12 | HADH | 10 | 3.303 | 0.3303 | 1.4513 |
| 1c1y | RAF1 | 9 | 2.969 | 0.3299 | 0.11631 |
| 1ryf | RAC1 | 11 | 3.625 | 0.3295 | 0.867649 |
| 1jk3 | MMP12 | 7 | 2.304 | 0.3291 | -0.722078 |
| 1cm8 | MK12 | 9 | 2.946 | 0.3274 | 0.0522935 |
| 3bc3 | CTSL | 8 | 2.62 | 0.3274 | 0.555471 |
| 1xtj | DDX39B | 9 | 2.936 | 0.3263 | -0.105495 |
| 2c6c | FOLH1 | 8 | 2.597 | 0.3247 | -0.213661 |
| 1q91 | NT5M | 9 | 2.909 | 0.3232 | -0.374678 |
| 1hfc | MMP1 | 8 | 2.57 | 0.3213 | -0.462367 |
| 2f4j | ABL1 | 8 | 2.569 | 0.3212 | -0.343178 |
| 1gs4 | ANDR | 8 | 2.568 | 0.3209 | -0.749071 |
| 1qbk | RAN | 11 | 3.518 | 0.3199 | 1.27428 |
| 1yvl | STAT1 | 8 | 2.556 | 0.3195 | -0.526626 |
| 1gre | GSR | 9 | 2.875 | 0.3194 | -0.0813311 |
| 1pt9 | NNT | 8 | 2.551 | 0.3189 | -0.423138 |
| 1g1t | LYAM2 | 9 | 2.869 | 0.3187 | -0.086106 |
| 2rap | RAP2A | 10 | 3.176 | 0.3176 | 0.713773 |
| 1uw5 | PITPNA | 7 | 2.216 | 0.3165 | -0.472124 |
| 1b0f | ELNE | 9 | 2.84 | 0.3155 | -0.409816 |
| 1gzu | NMNAT1 | 7 | 2.189 | 0.3128 | -1.36484 |
| 1nus | NMNAT3 | 9 | 2.805 | 0.3116 | -0.3599 |
| 1qzy | AMPM2 | 9 | 2.804 | 0.3116 | 0.255714 |
| 1yz3 | PNMT | 11 | 3.419 | 0.3108 | 0.588828 |
| 1hna | GSTM2 | 10 | 3.051 | 0.3051 | -0.159867 |
| 1tfg | TGFB2 | 9 | 2.738 | 0.3042 | -0.289912 |
| 1h7u | PMS2 | 9 | 2.705 | 0.3006 | 0.0493099 |
| 3ctq | MAPK14 | 8 | 2.392 | 0.299 | -0.111192 |
| 3ccz | HMDH | 8 | 2.391 | 0.2989 | -0.856511 |
| 1hi3 | RNASE2 | 9 | 2.686 | 0.2984 | -0.573535 |
| 1cm0 | KAT2B | 9 | 2.664 | 0.2961 | -0.452458 |
| 2c6q | GMPR2 | 10 | 2.958 | 0.2958 | -0.0513401 |
| 3eyl | XIAP | 8 | 2.365 | 0.2956 | -0.263156 |
| 1fw1 | GSTZ1 | 10 | 2.945 | 0.2945 | 0.0190493 |
| 1r55 | ADA33 | 10 | 2.932 | 0.2932 | -0.277978 |
| 1pkw | GSTA1 | 9 | 2.634 | 0.2927 | -0.518024 |
| 1h0c | SPYA_ | 7 | 2.047 | 0.2924 | -1.62124 |
| 1sl4 | CD209 | 8 | 2.337 | 0.2922 | -0.669988 |
| 1jvd | UAP1 | 11 | 3.199 | 0.2908 | 0.410811 |
| 1pj2 | MAOM | 10 | 2.888 | 0.2888 | 0.432244 |
| 1rhu | CASP3 | 9 | 2.593 | 0.2881 | -0.123942 |
| 1md4 | GSTP1 | 10 | 2.865 | 0.2865 | -0.2558 |
| 1q0b | KIF11 | 10 | 2.852 | 0.2852 | -0.301923 |
| 1q5h | DUT | 10 | 2.806 | 0.2806 | -0.581791 |
| 1p9a | GP1BA | 8 | 2.235 | 0.2793 | -1.05402 |
| 1yzg | Arl5b | 11 | 3.069 | 0.279 | -0.0756889 |
| 1b55 | BTK | 12 | 3.281 | 0.2734 | 0.0418257 |
| 1joc | EEA1 | 10 | 2.725 | 0.2725 | -0.634025 |
| 1ek6 | GALE | 9 | 2.443 | 0.2715 | -0.55074 |
| 1gbn | OAT | 8 | 2.172 | 0.2715 | -1.33856 |
| 1tdi | GSTA3 | 9 | 2.441 | 0.2712 | -1.0875 |
| 1ywn | VGFR2 | 9 | 2.432 | 0.2702 | -0.814726 |
| 1pr9 | DCXR | 10 | 2.679 | 0.2679 | 0.124456 |
| 1m7b | RND3 | 10 | 2.679 | 0.2679 | -0.570664 |
| 1dug | FIBG | 11 | 2.922 | 0.2656 | -0.424039 |
| 1dan | FA7 | 12 | 3.175 | 0.2646 | 0.173714 |
| 1r1j | NEP | 8 | 2.115 | 0.2644 | -1.5882 |
| 1nue | NDKB | 10 | 2.628 | 0.2628 | -0.700509 |
| 1nst | NDST1 | 9 | 2.362 | 0.2625 | -0.933536 |
| 1z6t | APAF | 11 | 2.885 | 0.2623 | -0.493936 |
| 1k6m | Pfkfb1 | 11 | 2.872 | 0.2611 | -0.458752 |
| 1ipb | EIF4E | 10 | 2.567 | 0.2567 | -0.886319 |
| 1c9h | FKB1B | 9 | 2.291 | 0.2545 | -0.957672 |
| 1xw6 | GSTM1 | 10 | 2.522 | 0.2522 | -0.936751 |
| 1h9o | P85A | 8 | 2.017 | 0.2522 | -1.16516 |
| 1bmq | CASP1 | 11 | 2.774 | 0.2521 | -0.49784 |
| 1gw6 | LKHA4 | 9 | 2.24 | 0.2489 | -1.34602 |
| 2c72 | AOFB | 14 | 3.457 | 0.2469 | 1.58847 |
| 2b4y | SIRT5 | 14 | 3.385 | 0.2418 | 0.44962 |
| 1yb5 | QOR | 10 | 2.415 | 0.2415 | -0.16117 |
| 1zkk | SETD8 | 11 | 2.654 | 0.2413 | 0.270734 |
| 1xmm | DCPS | 11 | 2.632 | 0.2393 | -0.774306 |
| 1ivh | IVD | 12 | 2.836 | 0.2363 | -0.0230137 |
| 1pbk | FKBP3 | 11 | 2.464 | 0.224 | -0.764734 |
| 1z6z | SPR | 14 | 2.983 | 0.2131 | -0.0599031 |
| 1u32 | PPP1CC | 11 | 2.205 | 0.2004 | -1.33909 |
| 1i5r | HSD17B1 | 16 | 3.2 | 0.2 | -0.0416842 |
